# Resveratrol Sensitizes Selectively Thyroid Cancer Cell to 131-Iodine Toxicity

**DOI:** 10.1155/2014/839597

**Published:** 2014-09-03

**Authors:** Seyed Jalal Hosseinimehr, Seyed Amir Hossein Hosseini

**Affiliations:** Department of Radiopharmacy, Faculty of Pharmacy, Pharmaceutical Sciences Research Center, Mazandaran University of Medical Sciences, Sari, Iran

## Abstract

*Background.* In this study, the radiosensitizing effect of resveratrol as a natural product was investigated on cell toxicity induced by ^131^I in thyroid cancer cell. *Methods.* Human thyroid cancer cell and human nonmalignant fibroblast cell (HFFF2) were treated with ^131^I and/or resveratrol at different concentrations for 48 h. The cell proliferation was measured by determination of the percent of the survival cells using 3-(4,5-dimethylthiazol-2-yl)-2,5-diphenyltetrazolium bromide (MTT) assay. *Results.* Findings of this study show that resveratrol enhanced the cell death induced by ^131^I on thyroid cancer cell. Also, resveratrol exhibited a protective effect on normal cells against ^131^I toxicity. *Conclusion.* This result indicates a promising effect of resveratrol on improvement of cellular toxicity during iodine therapy.

## 1. Introduction

Radioiodine-131 (^131^I) as a radioactive iodine is widely used for treatment of patients with thyroid diseases, such as thyroid cancer and Graves' disease. It emits beta particles and has a physical half-life of 8.02 days [1]. DNA damage and chromosomal breaks are main reasons for cell damage and death. Reactive oxygen species (ROS) are generated by ^131^I [2, 3]; these toxic products can attack critical macromolecules, such as DNA, leading to cell damage and death [4, 5]. However, ^131^I concentrates at high level in thyroid tissue with a high target to nontarget ratio which is perfect for thyroid cancer therapy; it has side effects, such as sialadenitis, haematological depression, xerostomia, and radiation thyroiditis [6–11]. Several studies have shown that genetic damage is increased in patients after ^131^I therapy, with the frequency of micronuclei being elevated [12–14]. The occurrence of secondary malignancies and leukaemia might increase with higher radioactive iodine doses [7]. Thus, protection of normal cells may mitigate side effects induced by ^131^I. Resveratrol is a natural polyphenol compound that is found in fruits, such as grapes. Several pharmacological properties were reported for resveratrol, such as neuroprotective, chemosensitive, anti-inflammatory, anticancer, antitumourigenic, chemopreventive, and antioxidant actions [15–20]. Recently we showed that resveratrol protected genotoxicity induced by ^131^I on normal human lymphocytes; it significantly reduced the DNA damage induced by ^131^I in vitro [21]. Sebastià reported that resveratrol protected human lymphocytes against genotoxicity induced by gamma radiation [22]. Scavenging of free radicals is proposed as the main mechanisms for protective effects of resveratrol [23, 24]. However, resveratrol exhibited protective effects on cellular toxicity induced by beta particle on normal cells; its effect is unclear on thyroid cancer cell during iodine-131 therapy. To further explore the beneficial effects of resveratrol, the aim of this study was to investigate its therapeutic effects on cell death induced by ^131^I in thyroid human cancer and human nonmalignant fibroblast cells in vitro.

## 2. Materials 

### 2.1. Chemicals

Resveratrol (RSV) and 3-[4,5-dimethylthiazol-2-yl]-2,5-diphenyltetrazolium bromide (MTT) was purchased from Sigma (USA). ^131^I-Na in sterile solution was prepared by AEOI, Tehran, Iran, and was used freshly.

### 2.2. Cell Culture

Human thyroid cancer (Thr.C1-PI 33) and human nonmalignant skin fibroblast (HFFF2) cells were got from the Pasture Institute of Iran. These cells were cultured at 37°C and 5% CO_2_ in Roswell Park Memorial Institute (RPMI) 1640 medium (Gibco, Paisley, UK) supplemented with 10% fetal bovine serum (FBS) and 100 *μ*g/mL penicillin-streptomycin (Gibco). Experiments on cells were performed in the exponential growth phase.

### 2.3. Cell Antiproliferation Assay

Untreated and treated thyroid cancer and HFFF2 cells were subjected to cell proliferation assay using MTT to quantify the metabolic activity to cleave tetrazolium salts [25, 26]. Cells (20,000) were seeded in 96-well plates. After 24 h incubation, cells were treated with various concentrations of RSV (0.5, 5, 10, and 50 *μ*g/mL) and incubated for 48 h at 37°C and 5% CO_2_. RSV was dissolved in ethanol and diluted with medium. After 48 hours of culture, 20 *μ*L MTT (5 mg/mL in phosphate buffer saline) was added to each well, and culturing was continued for 4 hours. Then, culture supernatant was discarded and replaced by DMSO, and the cell plates were shaken for 10 minutes. The absorbance of every culture well was read on an ELISA Reader (Bioteck, USA).

### 2.4. Irradiation Protocol

Cells were seeded in 96-well plates. After 24 h incubation, cells were treated with various concentrations of RSV (0.5, 5, 10, and 50 *μ*g/mL) and incubated for 2 h at 37°C and 5% CO_2_. After incubation, the solution of ^131^I was added at dose 10 *μ*Ci in 100 *μ*L to each well and incubated for 48 h. MTT assay was performed according to above protocol.

### 2.5. Statistical Analysis

Data were presented as mean ± standard deviation (SD) of three independent experiments. Data were compared with student *t*-test and the differences were considered significant if the *P* value < 0.05.

## 3. Results

### 3.1. Effect of Resveratrol on Cell Proliferation in Thyroid Cancer and HFFF2

**Figure 1:**
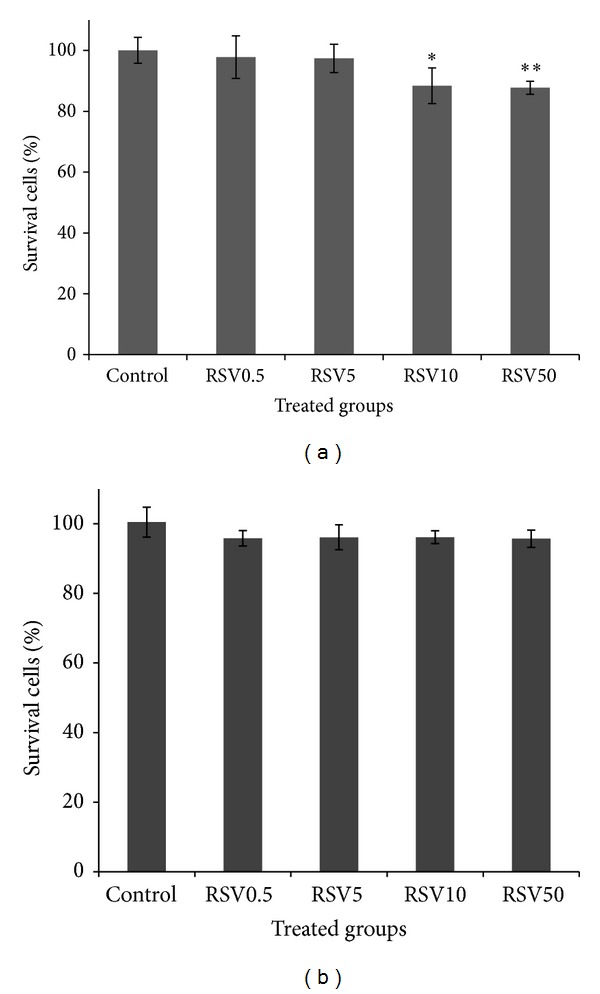
Effect of resveratrol (RSV) at different concentrations (0.5, 5, 10, and 50 *μ*g/mL) on thyroid cancer cells (a) and nonmalignant fibroblast cell (HFFF2) (b). Cell proliferation was assayed with MTT test (*n* = 4). *P < 0.05, comparing RSV10 with control. **P < 0.05, comparing RSV50 with RSV5.

Effects of RSV on cell proliferation in thyroid cancer and HFFF2 were determined by MTT assay. Thyroid cell proliferation was significantly inhibited by RSV at concentrations 10 and 50 *μ*g/mL (*P* < 0.02). A statistical difference between concentrations of RSV at doses 5 and 50 *μ*g/mL was observed. RSV exhibited a reduction of 12% in cellular growth in thyroid cells when cells were treated with 10 and 50 *μ*g/mL of RSV.  Figure 1(a) shows the percentage of cell proliferation in the thyroid cancer cells treated by RSV. In the comparison of cancer cell, human nonmalignant fibroblast cell (HFFF2) was used for any effect of RSV on cell proliferation. RSV did not cause significant cellular toxicity in HFFF2 cell (Figure 1(b)).

### 3.2. Effect of Resveratrol and ^131^I on Cell Proliferation in Thyroid Cancer and HFFF2

**Figure 2:**
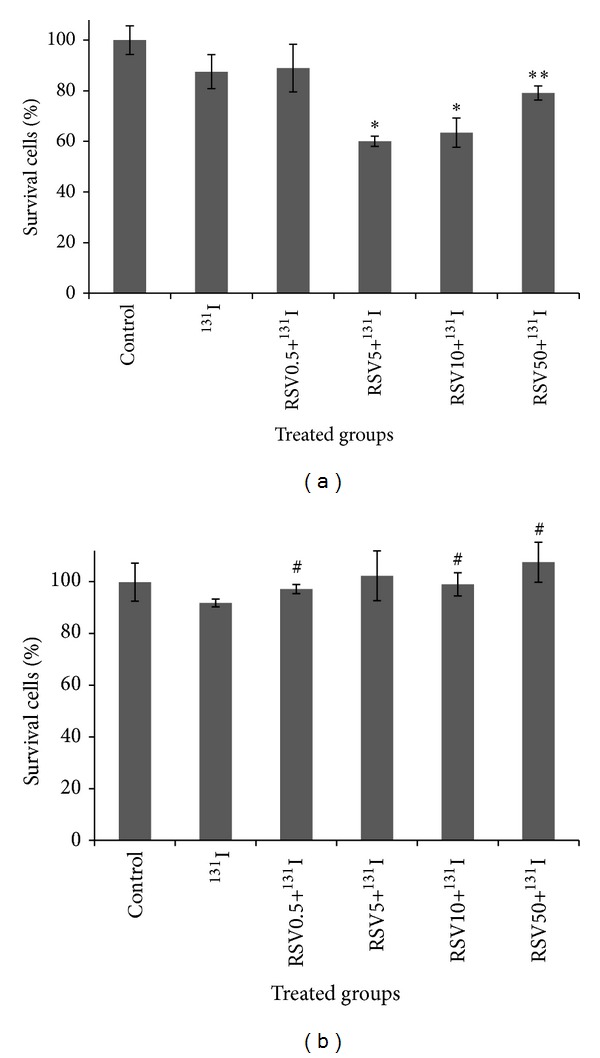
Effect of resveratrol (RSV) at different concentrations (0.5, 5, 10, and 50 *μ*g/mL) in combination with ^131^I on thyroid cancer cells (a) and nonmalignant fibroblast cell (HFFF2) (b). Cell proliferation was assayed with MTT test (*n* = 4). *P < 0.05, comparing RSV5 and RSV10 with ^131^I.  **Nonsignificant, comparing RSV50 and ^131^I. ^#^ *P* < 0.05, comparing RSV0.5, 10, and 50 with ^131^I.

Figure 2 shows the combination effect of RSV and 131-iodine on percentage of cell proliferation in control, RSV-pretreated, and/or ^131^I in thyroid cancer and HFFF2 cells. ^131^I significantly reduced survival rate in thyroid cancer cell by 87%. Thyroid cancer cell proliferation was significantly reduced in RSV treated groups. RSV significantly reduced percentage of cell survival to 60% and 63% at concentrations 5 and 10 *μ*g/mL, respectively. These results indicate that RSV has synergetic effects with ^131^I on inhibition of cell growth on thyroid cancer cell. A radiosensitive effect by RSV in thyroid cancer cells treated with ^131^I was observed. It is interesting that RSV was not shown any enhancement of toxicity on HFFF2 cell in combination with ^131^I. RSV exhibited an increase of cell growth in combination with ^131^I in HFFF2 cells at concentrations of 0.5, 10, and 50 *μ*g/mL when these RSV treated groups were compared to ^131^I alone (*P* < 0.05).

## 4. Discussion

In this study, RSV exhibited a radiosensitizing effect on thyroid cancer cell; it reduced cell growth in combination with ^131^I. RSV increased cell growth in nonmalignant fibroblast cell (HFFF2) treated with ^131^I. Then RSV exhibited a radiosensitive effect on cancer cell and radioprotective effect on normal cell. This dual effect of RSV is dependent on type of cell. Radioactive iodine-131 (^131^I) is widely used for the treatment of thyroid-related diseases. ^131^I is taken up almost exclusively by thyroidal tissue, and high-dose radioiodine treatment is associated with limited side effects such as sialadenitis and xerostomia. Patients suffer from these side effects. Pharmacological treatment can be a promising strategy for protecting patients from side effects induced by ^131^I therapy [3]. Recently we showed RSV significantly protected human lymphocytes from genotoxicity induced by ^131^I. RSV reduced micronuclei frequency in lymphocytes in combination with ^131^I [21]. In this study we tried to evaluate the effects of RSV on thyroid cancer cells, because it is hypothesised that RSV may have a protective effect on thyroid cancer cells, which will be contraindicated in thyroid cancer therapy with ^131^I. Our results indicate that RSV has radiosensitizing effects on thyroid cancer cell and radioprotective effects on normal cells against cellular toxicity induced by ^131^I. These results are promising for using of this natural product in ^131^I therapy in patients. Resveratrol has been shown to have several biological properties such as antioxidant activity, induction apoptosis in cancer cells, and inflammation [27–29]. RSV induced apoptosis in several cancer cells such as human colorectal and bladder cancers. This effect was through activation of caspase and regulation of the Akt/Bcl-2 signaling pathway [30, 31]. RSV sensitized colon cancer cell lines to 5-fluorouracil treatment on the increase of apoptotic effect and exhibited stronger antitumor effect [32]. In our study, RSV significantly sensitized thyroid cancer cell to ^131^I at concentrations 5 and 10 *μ*g/mL, while this cellular toxicity was not increased at higher concentration 50 *μ*g/mL. RSV maximally enhanced cell death induced by ^131^I at concentration 5 *μ*g/mL. RSV probably has tumor cell toxicity through activation or inhibition of cellular signal pathways, which was established in other studies. RSV induced apoptosis in thyroid carcinoma cells; it acts via a Ras-MAPK kinase pathway to increase p53 expression [28, 33–35]. RSV suppressed anaplastic thyroid carcinoma cell growth via S-phase cell-cycle arrest and apoptosis; it induced functional Notch1 protein expression and activated the pathway by transcriptional regulation [36]. Also, resveratrol increased iodide trapping in FRTL-5 cells, iodide influx, and rNIS protein level even in the absence of TSH. These mechanisms may contribute to enhancement of cell toxicity through increasing of ^131^I uptake by thyroid cancer cells [37]. Antioxidant activity against cellular oxidative stress is one of the main mechanisms related to protection of RSV. RSV directly scavenges reactive oxygen species produced by oxidative stress and it is probably related to the presence of hydroxyl groups on the chemical structure of RSV [38, 39]. Resveratrol strongly prevented C6 cells from H_2_O_2_-induced toxicity by modulating glial, oxidative, and inflammatory responses. Resveratrol increased heme oxygenase 1 (HO1) expression and extracellular GSH content [40]. Ionizing radiation produces free radicals that damages macromolecules such as DNA leading to cell death in normal tissues. RSV protected normal cells against genotoxicity induced by ionizing radiation. Antioxidant activity is a main mechanism for radioprotective of RSV in normal cells [21, 41]. Also resveratrol inhibits IL-1*β* expression induced by radiation via the activation of Sirt1; this mechanism participates in radioprotection [42].

Our findings indicate that resveratrol is a promising natural product in patients on radioiodine therapy; it sensitizes thyroid cancer cell to ^131^I. Also, RSV is an effective protective agent on normal cells against toxicity induced by radioiodine therapy. With these two beneficial actions, RSV may improve the treatment of patients with thyroid cancer during radioiodine therapy.
